# Controlled Defects of Zinc Oxide Nanorods for Efficient Visible Light Photocatalytic Degradation of Phenol

**DOI:** 10.3390/ma9040238

**Published:** 2016-03-28

**Authors:** Jamal Al-Sabahi, Tanujjal Bora, Mohammed Al-Abri, Joydeep Dutta

**Affiliations:** 1 Department of Chemical and Petroleum Engineering, College of Engineering, Sultan Qaboos University, PO Box 33, PC 123, Al-Khoudh, Oman; jamal@squ.edu.om; 2 Chair in Nanotechnology, Water Research Center, Sultan Qaboos University, PO Box 17, PC 123, Al-Khoudh, Oman; tanujjal@squ.edu.om; 3 Functional Materials Division, Materials and Nanophysics, ICT School, KTH Royal Institute of Technology, Isafjordsgatan 22, Kista Stockholm SE-164-40, Sweden

**Keywords:** zinc oxide, surface defect, oxygen vacancy, annealing, phenol, photocatalysis

## Abstract

Environmental pollution from human and industrial activities has received much attention as it adversely affects human health and bio-diversity. In this work we report efficient visible light photocatalytic degradation of phenol using supported zinc oxide (ZnO) nanorods and explore the role of surface defects in ZnO on the visible light photocatalytic activity. ZnO nanorods were synthesized on glass substrates using a microwave-assisted hydrothermal process, while the surface defect states were controlled by annealing the nanorods at various temperatures and were characterized by photoluminescence and X-ray photoelectron spectroscopy. High performance liquid chromatography (HPLC) was used for the evaluation of phenol photocatalytic degradation. ZnO nanorods with high surface defects exhibited maximum visible light photocatalytic activity, showing 50% degradation of 10 ppm phenol aqueous solution within 2.5 h, with a degradation rate almost four times higher than that of nanorods with lower surface defects. The mineralization process of phenol during degradation was also investigated, and it showed the evolution of different photocatalytic byproducts, such as benzoquinone, catechol, resorcinol and carboxylic acids, at different stages. The results from this study suggest that the presence of surface defects in ZnO nanorods is crucial for its efficient visible light photocatalytic activity, which is otherwise only active in the ultraviolet region.

## 1. Introduction

Phenol and its derivatives are widely used in several manufacturing industries and can find their way into the environment, polluting the ground water or surface water resources [1]. Phenol can accumulate over several years in the presence of the point source if not treated properly, which can lead to a serious threat to human health [2] and can adversely affect sustainable development of societies and aquatic life [3]. Phenolic compounds have been found in different sewage sludge, influent and effluent of wastewater, river water and soil [4,5,6,7]. The compounds 2-nitrophenol, 4-nitrophenol and 2.4-dinitrophenol, ranging from 0.1 to 5.0 µg/L, have been reported to be present in the Ebro river in Spain [7], while up to 40 mg/L in river water was reported due to the dispersal of wastewater from the petrol industry [8]. Physicochemical methods including adsorption using activated carbon, biological treatments and advanced oxidation processes (AOPs) are generally used for the degradation of phenolic compounds in water. In the quest for purifying polluted water, photocatalysis has become a popular choice in recent years, and it is an effective process for converting organic pollutants, such as phenol, into harmless products such as mineral acids, water and carbon dioxide [9,10]. Light, upon absorption in the photocatalyst, typically a semiconductor material, generates radicals that can degrade organic pollutants. Most of the widely used photocatalysts, e.g., titanium dioxide (TiO_2_), tin dioxide (SnO_2_) and zinc oxide (ZnO), are wide-bandgap metal oxides, and thus high energy ultraviolet (UV) irradiation is needed to activate them, making it difficult for large-scale, economically viable photocatalytic applications. Therefore, research is now focusing on using solar energy for the photocatalytic degradation of organic pollutants in water. The amount of UV irradiation in solar light is about 5% of the spectrum, while 45% of sunlight is in the visible light region [11]. In order to efficiently utilize solar energy, photocatalysts need to be modified to make them active in the visible region of the solar spectra [12].

In order to improve visible light absorption in wide-bandgap metal oxide semiconductors, metal and non-metal doping, plasmon coupling, band-matching with two semiconductors and self-doping by crystal defects have been discussed in the literature. Doping by introducing metal and non-metal elements shows improvements in visible light absorption in wide-bandgap semiconductors [13,14,15]. For example, when ZnO is doped to Cu and Ag, it improves the photocatalytic activity of ZnO under visible light irradiation [16,17]. The improvement of photocatalytic activity of ZnO is also observed by effective charge separation through the coupling of a metal deposit (nanoparticles) on the photocatalyst surface [18,19,20,21]. Furthermore, the mixed composite of two semiconductors [22,23,24] has been shown to be effective in efficient charge separation upon visible light absorption. Visible light absorption can also be increased through defect engineering of the photocatalyst surface, sometimes called self-doping [25,26]. Increasing or controlling the crystal defects in the semiconductor also increases the range of visible light absorption of the material, making it active under solar light [27]. Many studies showed promising improvement in the photocatalytic activities of metal oxide semiconductors by creating defects on the surface of the catalyst. It was shown that oxygen vacancies can be controlled by annealing ZnO nanoparticles or nanorods at elevated temperatures, enhancing the photocatalytic degradation of organic contaminants [28,29].

In this work, we study the defect-induced photocatalytic activity of ZnO nanorods with a careful comparison of the surface defect states of the ZnO nanorods using phenol as a test contaminant. Density of the surface defect states in ZnO nanorods was controlled by annealing the nanorods in ambient atmosphere at moderate temperatures and their influence on the visible light photocatalytic activity of the ZnO nanorods was explored.

## 2. Results and Discussion

**Figure 1:**
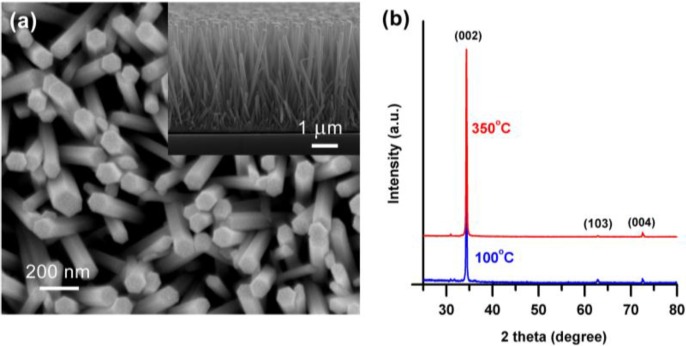
(**a**) SEM micrograph of ZnO nanorods grown on glass substrate using a microwave-assisted hydrothermal process. The sample was annealed at 350 °C in air for 1 h, followed by the growth. Inset shows the cross-sectional view of the vertically aligned ZnO nanorods; (**b**) XRD patterns of ZnO nanorods annealed at 100 and 350 °C in order to vary the concentrations of surface defects in the nanorods.

Figure 1a shows the SEM micrographs of vertically aligned hexagonal wurtzite ZnO nanorods grown on glass substrates annealed in air at 350 °C. The average length of the ZnO nanorods was found to be ~4.3 ± 0.2 µm (estimated from the cross-sectional view of the samples as shown in the inset) and the average diameter was 100 ± 10 nm. It should be noted that no morphological changes were observed between the samples annealed at 100 °C (data not shown here) and 350 °C, respectively. XRD patterns of the ZnO nanorods annealed at 100 and 350 °C are shown in Figure 1b. Both the ZnO nanorod samples showed characteristic XRD peaks of hexagonal wurtzite crystal structure confirmed from JCPDS card No. 01-070-8070. No variations in the XRD patterns were observed between the ZnO nanorods annealed at different temperatures. The strongest XRD peak at 34.35° indicated the preferential orientation of the ZnO nanorods along the (002) crystal plane [30].

**Figure 2:**
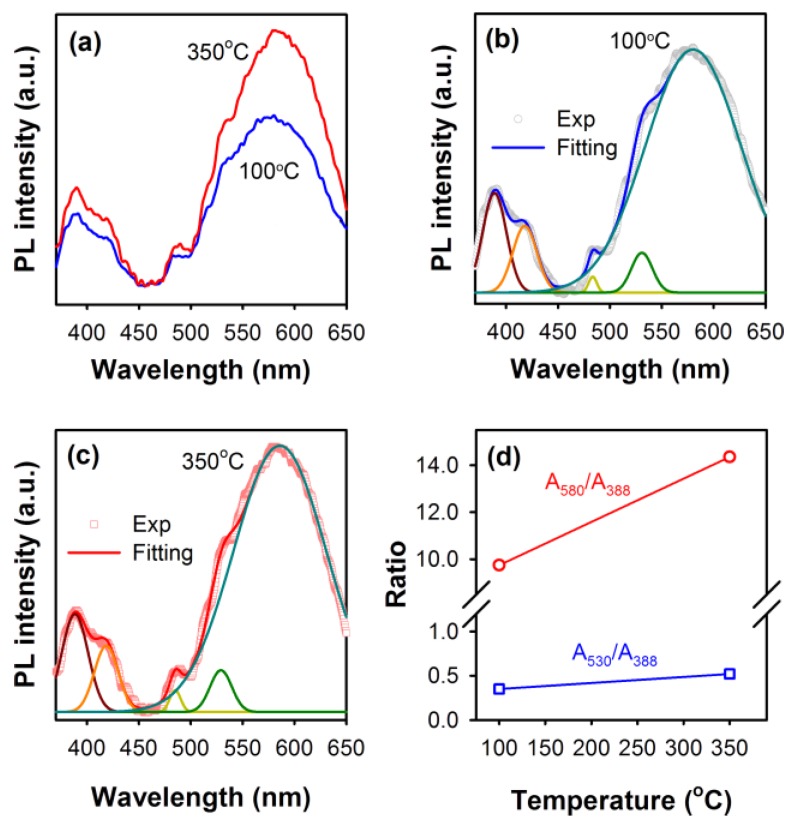
(**a**) Room temperature photoluminescence (PL) spectra of ZnO nanorods annealed at 100 and 350 °C (Excitation: 350 nm). Both the PL spectra were found to be composed of five Gaussian components, which are shown in (**b**) and (**c**). The relative concentrations of surface-situated oxygen vacancy states in the annealed ZnO nanorods, represented by PL components at 530 and 580 nm (V_o_^+^ and V_o_^++^, respectively), are shown in (**d**) as the ratio of area under the PL bands to the area under the band-to-band transition observed at 388 nm.

Surface defects in the ZnO nanorods modulated by annealing them at two different temperatures were then characterized by photoluminescence (PL) and X-ray photoelectron spectroscopy (XPS) techniques. The room temperature PL spectra of ZnO nanorods annealed at 100 and 350 °C are shown in Figure 2a, where emissions in both the UV and visible regions are observed. The PL bands in the high energy region are found to be composed of two major Gaussian components, peaking at around 388 nm (~3.2 eV) and 418 nm (~2.9 eV), respectively, as shown in Figure 2b,c, representing the band-to-band transitions in ZnO and a radiative transition from a defect state situated near the conduction band (CB) of ZnO, respectively. Deep-level zinc interstitial (Zn_i_) defects, typically found at ~0.22 eV below the CB of ZnO, are reported as the possible origin of the violet emission from ZnO [31].

The broad emissions from the ZnO nanorods observed in the green-yellow (GY) region, typically attributed to the surface defects in ZnO nanostructures, were also found to be composed of three major Gaussian components centered at 483, 530 and 580 nm. The small 483 nm component can be related to the zinc vacancy states (V_Zn_), whereas the 530 and 580 nm PL bands are due to the singly and doubly charged oxygen vacancy states (V_o_^+^ and V_o_^++^), respectively [32,33]. As shown in Figure 2d, upon annealing the ZnO nanorods at 350 °C, the relative concentration of oxygen vacancies near the surface, measured as the ratio of the area under the Gaussian components to the area under the band-to-band transition at 388 nm, was found to increase compared to the 100 °C annealed ZnO nanorods, indicating that oxygen vacancy states diffuse towards the surface upon annealing at higher temperature due to the increase in the self-diffusion coefficient of the oxygen vacancy states [31]. Further, since in ZnO wurtzite crystal the axial ratio of the oxygen *hcp* lattice (*c*/*a* ~1.606) is slightly smaller than ideal (*c*/*a* ~1.633), the out-of-plane diffusion of defects is dominant, allowing the oxygen vacancies to migrate towards the surface of the nanorods [34]. As a result, upon annealing the nanorods at 350 °C, the relative concentration of oxygen vacancy sites gradually increases near the surface.

**Figure 3:**
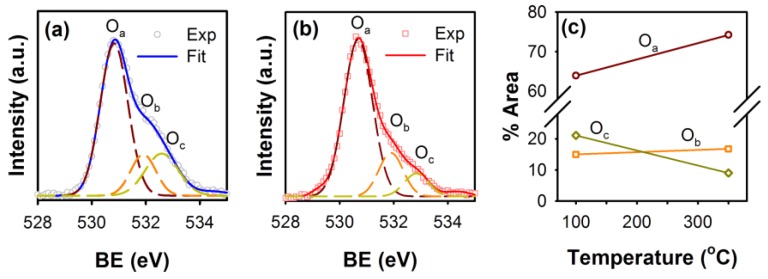
Oxygen 1s XPS spectra of ZnO nanorods annealed at (**a**) 100 °C and (**b**) 350 °C. The O1s spectra were coherently fitted with three Gaussian peaks (O_a_, O_b_ and O_c_); (**c**) Percentage variations in the area under O_a_, O_b_ and O_c_ representing the chemical states of oxygen at two different annealing temperatures of the ZnO nanorods.

In order to further verify the chemical states of oxygen near the surface of the annealed ZnO nanorods, we carried out XPS measurements on the ZnO nanorods annealed at 100 and 350 °C. Figure 3 shows the asymmetric O1s XPS spectra of the ZnO nanorods annealed at different temperatures. Both asymmetric O1s XPS spectra were coherently fitted by three Gaussian components, centered at 530.6 eV (O_a_), 531.9 eV (O_b_) and 532.6 eV (O_c_), respectively, as shown in Figure 3a,b. The O_a_ peak on the lower binding energy side of the O1s spectrum can be attributed to the O_2_^−^ ions which are surrounded by zinc atoms with the full supplement of nearest-neighbor O_2_^−^ ions [34]. Thus, the O_a_ peak of the O1s spectrum can be attributed to the Zn–O bonding. The O_c_ peak towards the higher binding energy at 532.6 eV is usually associated with the chemisorbed or dissociated oxygen or OH species on the surface of ZnO, such as adsorbed H_2_O or adsorbed O_2_ [35,36]. The middle component at the binding energy 531.9 eV (O_b_) of the O1s spectra is related to the O_2_^−^ ions that are in oxygen-deficient regions within the ZnO matrix (oxygen vacancies) [37,38]. Therefore, changes in the area under these peaks upon annealing can be correlated to the variations in the chemical states of oxygen at the surface of the ZnO nanorods.

The area of the peak O_a_ was found to increase from 63.8% to 74.3% when ZnO nanorods were annealed at 350 °C, indicating the improvement in the Zn–O bonding in ZnO nanorods upon annealing. In this regard, it has been shown that, at annealing temperatures above 300 °C, the Zn–O bonding stoichiometry can be enhanced [39,40]. Also, the area under the O_c_ peak was observed to decrease, suggesting the reduction of the surface-adsorbed OH species in the ZnO nanorods upon annealing. However, the area under the O_b_ peak related to the oxygen vacancies was observed to increase from 14.9% to 16.8% when nanorods were annealed at 350 °C, indicating the accumulation of oxygen vacancy states near the surface of the ZnO nanorods, confirming the diffusion of oxygen vacancies towards the surface of the ZnO nanorods upon annealing them at 350 °C resulting in the increase in the GY PL band intensity compared to the 100 °C annealed nanorods, as observed in Figure 2a.

**Figure 4:**
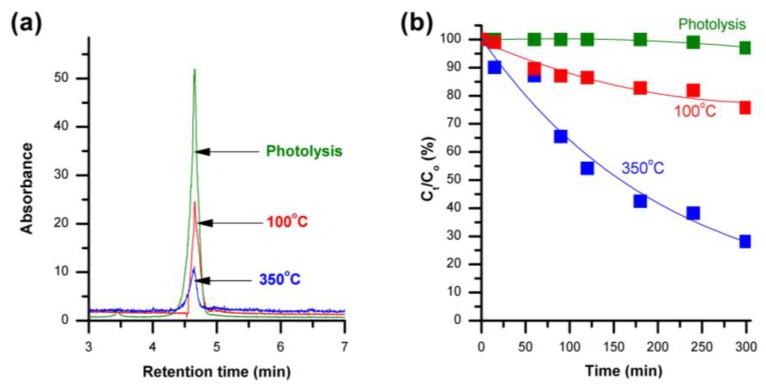
(**a**) High performance liquid chromatography (HPLC) chromatograms representing the phenol peak at retention time 4.66 min after 300 min of photocatalytic degradation with and without the ZnO nanorods; (**b**) Visible light photocatalytic degradation kinetics of phenol (initial concentration: 10 ppm) with and without the ZnO nanorods having different surface defect densities.

The ZnO nanorods with two different surface defect densities were then used to study the photocatalytic degradation of phenol under solar light irradiation. Figure 4a shows a schematic diagram of three chromatograms of phenol degradation after 300 min with and without the ZnO nanorods. The retention time for phenol under the given High performance liquid chromatography (HPLC) operating conditions was recorded at 4.66 min. The concentration of phenol at different times was estimated from the area under the phenol peak detected at 4.66 min.

When phenol was exposed to light only in the absence of ZnO nanorods, it showed a slight degradation of about 3% after 300 min, indicating that visible light has almost no effect on the degradation of phenol in aqueous medium. When ZnO nanorods with lower surface defects were used, only about 20% degradation of phenol was observed after 300 min (Figure 4b), showing a degradation rate constant of 1 × 10^−3^ min^−1^. In this regard, it has been reported earlier that the presence of surface defects in ZnO nanostructures increases the rate of electron-hole pair generation through increased sub-bandgap absorption, enhancing the photocatalytic activity of the material in the visible region [41,42]. Additionally, the increased surface defect density in the 350 °C annealed ZnO nanorods increases the number of active sites near the surface of the nanorods by acting as trap sites for the photo-generated electrons, allowing the photoactive charges to interact easily with the phenol molecules [41]. As a result, when ZnO nanorods with higher surface defects (annealed at 350 °C) were used, the photocatalytic degradation of phenol was further observed to enhance demonstrating the degradation rate constant (4.3 × 10^−3^ min^−1^) comparable to what has been reported in the literature using titanium dioxide (TiO_2_) nanoparticles and UV light irradiation [1], and it is more than four times that of the nanorods with lower defects.

**Figure 5:**
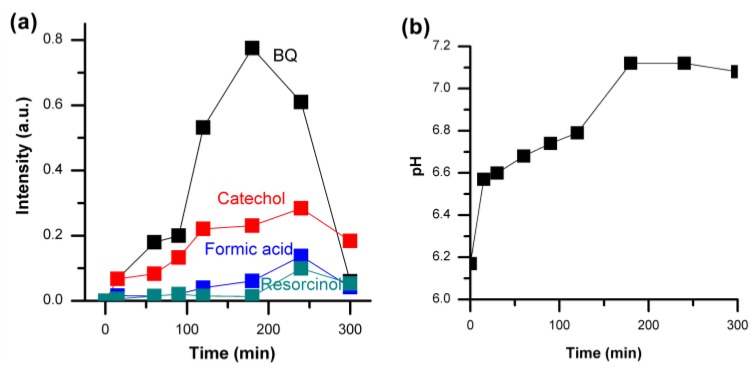
(**a**) Intermediate byproducts detected at various stages of photocatalytic degradation of phenol under visible light irradiation with ZnO nanorods as photocatalyst and (**b**) variations in the pH of the phenol solution during the degradation process.

In order to understand the detailed photocatalytic degradation process of phenol pathways, we have studied the formation of byproducts during the degradation of phenol. Degradation of phenol initially starts with the breakage of the O–H bonds, resulting in phenol giving up a proton (H^+^). It results in the formation of an anion stabilized by the aromatic ring [43]. Figure 5a shows the evolution of different reaction byproducts detected during the photocatalytic degradation of phenol using the ZnO nanorods with higher surface defects. Benzoquinone (BQ), catechol, resorcinol and formic acid were detected as the major byproducts of the phenol photocatalytic degradation under visible light irradiation. The intermediates that could be detected were benzoquinone, catechol, formic acid and resorcinol.

**Figure 6:**
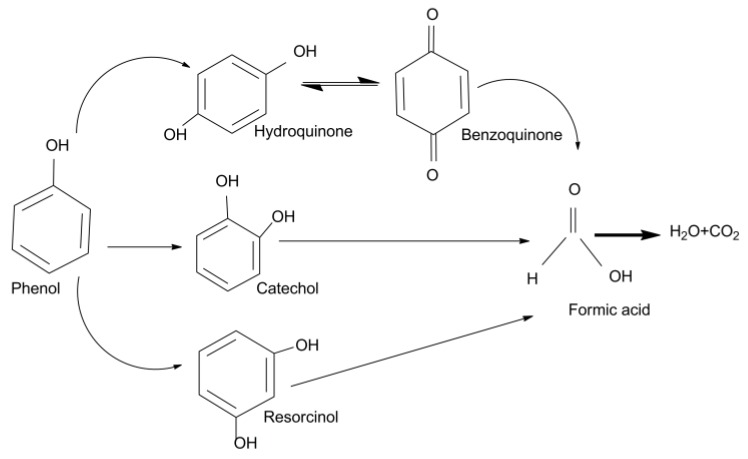
Photocatalytic degradation pathways of phenol under visible light irradiation in the presence of ZnO nanorods with high surface defects as photocatalysts depicting the complete mineralization process of phenol where O–H bonds are broken and resulting in the formation of multiple channels starting with hydroquinone, catechol and resorcinol. Benzoquinone is reversible with hydroquinone. The aromatic ring is opened and results in the formation of aliphatic compounds, such as mineral acids, water and carbon dioxide.

BQ forms readily upon photoirradiation in the presence of a photocatalyst, with the maximum reached within 180 min, and it is almost intractable in samples subjected to photocatalysis for 300 min. The formation of BQ at the early stage was also reflected from the pH of the phenol solution (Figure 5b) as it rapidly increased towards basic pH from 6.2 to 6.6, reaching a maximum pH of 7.2 at around 180 min where the BQ concentration was also at its peak. Catechol and resorcinol were observed at the early stages during the photodegradation, processing very low concentrations. The presence of both catechol and resorcinol were found to decrease beyond 200 min of photoirradiation and were almost undetectable after 300 min of photocatalytic reaction. The pH of the phenol solution was observed to shift slightly towards the acidic region due to the formation of formic acids after about 180 min of photocatalytic degradation during our experiments. Based on these results, the photocatalytic degradation pathway for phenol under visible light irradiation in the presence of ZnO nanorods with high surface defects is presented in Figure 6, showing the complete mineralization process of phenol. Hydroxyl radicals produced on the photocatalyst surface react with phenol to produce hydroquinone. Phenol produces phenoxy radicals that are in resonance with ortho- and para-position radical structures which lead to the formation of the three mesomeric forms of the radical, which reacts with hydroxyl radicals to form hydroquinone, benzoquinone, and catechol [43]. Hydroquinone reacts with OH^−^ to form benzoquinone. Upon extended photo-oxidation, the benzene ring can open due to continuous oxidation, leading to the formation of aliphatic compounds and ultimately mineralizing to form carbon dioxide (CO_2_) and water upon complete oxidation, which is schematically presented in Figure 6.

## 3. Materials and Methods

### 3.1. Preparation of Zinc Oxide Nanorods

The synthesis of ZnO nanorods was carried out using microwave-assisted hydrothermal process [44,45]. ZnO nanorods were grown using a two-step process: (a) deposition of a seed layer on the glass substrate and (b) growth of the nanorods. The glass substrates were first carefully cleaned using detergent, ethanol and followed by acetone and finally deionized (DI) water under sonication for 15 min each. For ZnO seeding process, a solution (10 mM) was prepared by dissolving zinc acetate dihydrate (Zn(CH_3_COO)_2_·2H_2_O; Merck, Billerica, MA, USA) in DI water. The ZnO seed layer was deposited by spraying the solution on the glass substrate placed on a hotplate at 350 °C. ZnO nanorods were then grown by horizontally dipping the seeded substrates in a mixture of equimolar concentrations of 20 mM zinc nitrate hexahydrate (Zn(NO_3_)_2_·6H_2_O; Sigma, Saint Louis, MO, USA) and hexamethylenetetramine (HMTA; Merck) solution in DI water and heating in a microwave oven at a power setting of 180 W for 45 min. The temperature of the growth solution was 90 °C. After 45 min the old growth solution was replaced with new solution and the cycle was repeated four more times. After that, obtained glass substrates containing ZnO nanorods were removed from the solution, rinsed thoroughly with DI water, and dried in an oven at 90 °C. In order to modulate the density of surface defects in the ZnO nanorods, some ZnO nanorod samples were annealed at 100 °C and rest were annealed at 350 °C in air for 1 h in a furnace (Carbolite CWF 1200, Derbyshire, UK).

### 3.2. Characterization

The morphology of the ZnO nanorods was investigated by field emission scanning electron microscopy (FESEM, JSM-7600F, JEOL, Tokyo , Japan, operated at 20 kV) and the crystal structure was evaluated using X-ray diffraction (XRD, Miniflex 600, Rigaku, Tokyo , Japan) with Cu Kα radiation in the scanning range from 20° to 80° in 0.02°/s steps. Photoluminescence (PL) spectra of the samples were collected by using fluorescence spectrometer (LS 55, Perkin Elmer, Waltham, MA, USA) at room temperature with 350 nm excitation wavelength (~3.54 eV). X-ray photoemission spectroscopy (XPS; Omicron Nanotechnology, Taunusstein, Germany) with a monochromatic Al Kα radiation (energy = 1486.6 eV) working at 15 kV was used to study the surface states of the ZnO nanorods. The obtained XPS spectra were calibrated with respect to the C1s feature at 284.6 eV. During the XPS measurements, ZnO samples were flooded with electrons to avoid surface charging during the XPS measurements.

### 3.3. Photocatalytic Activity Test

A phenol solution (10 ppm) was prepared in organic-free DI water. Amount of 3 mL of the phenol solution was put into 3.5 mL plastic cuvettes. The glass slide (dimensions of 2.5 cm × 0.9 cm) containing ZnO nanorods with different surface defect densities, that were previously prepared by using microwave-assisted hydrothermal process, were placed semi-vertically in the cuvettes and they were kept under stimulated solar light (AM 1.5 radiation, 1 kW/m^2^) obtained from a solar simulator (Sciencetech SS1.6 kW, London, ON, Canada). A control sample of 10 ppm phenol in DI water without any ZnO nanorods was also placed under the simulated solar light. The photocatalytic degradation was carried out for 300 min and phenol fractions (50 μL) were collected at regular intervals and degradation kinetics was studied by analyzing the phenol solution by using ultra performance liquid chromatography (UPLC, LC-30AD, Shimadzu, Tokyo, Japan) technique. Prior to the starting of the photocatalytic reactions, adsorption test was conducted in dark to attain an adsorption/desorption equilibrium of phenol with the ZnO nanorods and it was found that within 2 h of dipping into phenol solution the equilibrium was achieved. For all photocatalytic experiments, ZnO nanorod samples were soaked in phenol solution for 2 h prior to the photocatalysis experiments.

### 3.4. Analytical Method of Degradation Assay

The change in phenol concentration over time by using ZnO nanorods with different surface defect densities was monitored by ultra performance liquid chromatography (UPLC). An autosampler (SIL-30A, Shimadzu, Tokyo, Japan) was used for introducing 20 µL of the samples into the chromatograph. The analysis was carried out using a ODS hypersil column (3 µm particle size, 4.6 mm internal diameter (I.D.), 80 mm length, Hewlett Packard, Santa Clara, CA, USA) maintained at room temperature. The mobile phase was a mixture of water and methanol in a ratio 55:45 and the pH was adjusted to 3 using sulfuric acid. The flow rate of the mobile phase was 0.3 mL/min, while the recorded pressure was around 50 bars. The compounds of interest were spectrally scanned (200–400 nm) by using Diode Array detector (Prominence SPD-M20A, Shimadzu, Tokyo, Japan) and five wavelengths (210, 245, 270, 276 and 290 nm) were selected for the analysis. The photocatalytic degradation kinetics of phenol was then plotted as *C*_t_/*C*_o_ against visible light irradiation time, where *C*_t_ is the concentration of phenol at irradiation time “t” measured by HPLC and *C*_o_ is the initial concentration of phenol (10 ppm).

## 4. Conclusions

Zinc oxide nanorods were synthesized on glass substrates using a microwave-assisted hydrothermal process and the surface defects in the nanorods were modulated by annealing the nanorods in air at different temperatures. The PL and XPS analysis showed maximum surface defects for the 350 °Cannealed ZnO nanorods. The role of the surface defects on the visible light photocatalytic activity of ZnO nanorods was then evaluated by degrading phenol in aqueous medium. ZnO nanorods with maximum surface defect densities showed the highest photocatalytic degradation rate for phenol and degraded more than 72% of the phenol in just 5 h of solar light irradiation. The intermediate products formed during the phenol photocatalysis were studied and the complete mineralization pathway for the visible light photocatalytic degradation of phenol in the presence of ZnO nanorods is presented.

## References

[1] Choquette-Labbé M., Shewa W.A., Lalman J.A., Shanmugam S.R. (2014). Photocatalytic degradation of phenol and phenol derivatives using a nano-TiO_2_ catalyst: Integrating quantitative and qualitative factors using response surface methodology. Water.

[2] Ahmed A.B., Jibril B., Danwittayakul S., Dutta J. (2014). Microwave-enhanced degradation of phenol over Ni-loaded ZnO nanorods catalyst. Appl. Catal. B.

[3] Abdelouahab N., Ainmelk Y., Takser L. (2011). Polybrominated diphenyl ethers and sperm quality. Reprod. Toxicol..

[4] Niedan V., Pavasars I., Öberg G. (2000). Chloroperoxidase-mediated chlorination of aromatic groups in fulvic acid. Chemosphere.

[5] Fries E., Püttmann W. (2003). Occurrence and behavior of 4-nonylphenol in river water of Germany. J. Environ. Monit..

[6] Berryman D., Houde F., DeBlois C., O’Shea M. (2004). Nonylphenolic compounds in drinking and surface waters downstream of treated textile and pulp and paper effluents: A survey and preliminary assessment of their potential effects on public health and aquatic life. Chemosphere.

[7] Pocurull E., Marcé R.M., Borrull F. (1996). Determination of phenolic compounds in natural waters by liquid chromatography with ultraviolet and electrochemical detection after on-line trace enrichment. J. Chromatogr. A.

[8] Bruce R.M., Santodonato J., Neal M.W. (1987). Summary review of the health effects associated with phenol. Toxicol. Ind. Health.

[9] Fulekar M.H., Pathak B., Kale R.K., Fulekar M.H., Pathak B., Kale R.K. (2014). Nanotechnology: Perspective for environmental sustainability. Environment and Sustainable Development.

[10] Tao Y., Cheng Z.L., Ting K.E., Yin X.J. (2013). Photocatalytic degradation of phenol using a nanocatalyst: The mechanism and kinetics. J. Catal..

[11] Yoon T.P., Ischay M.A., Du J. (2010). Visible light photocatalysis as a greener approach to photochemical synthesis. Nat. Chem..

[12] Lavand A.B., Malghe Y.S. (2015). Synthesis, characterization and visible light photocatalytic activity of nitrogen-doped zinc oxide nanospheres. J. Asian Ceram. Soc..

[13] Hu Y., Chen G., Li C., Yu Y., Sun J., Dong H. (2015). Improved light absorption and photocatalytic activity of Zn, N-TiO_2−*x*_ rich in oxygen vacancies synthesized by nitridation and hydrogenation. New J. Chem..

[14] Devi V., Joshi B.C., Kumar M., Choudhary R.J. (2014). Structural and optical properties of Cd and Mg doped zinc oxide thin films deposited by pulsed laser deposition. J. Phys..

[15] Zhen Z., Ji-ling S., Jia-hong Z., Jian-she L. (2014). Optical properties and photocatalytic activity of Nd-doped ZnO powders. Trans. Nonferrous Met. Soc..

[16] Georgekutty R., Seery M.K., Pillai S.C. (2008). A highly efficient Ag-ZnO photocatalyst: Synthesis, properties, and mechanism. J. Phys. Chem. C.

[17] Rahimi R., Shokrayian J., Rabbani M. Photocatalytic removing of methylene blue by using of Cu-doped ZnO, Ag-doped ZnO and Cu, Ag-codoped ZnO nanostructures. Proceedings of the 17th International Electronic Conference on Synthetic Organic Chemistry.

[18] Ruiz Peralta M.D.L., Pal U., Zeferino R.S. (2012). Photoluminescence (PL) quenching and enhanced photocatalytic activity of Au-decorated ZnO nanorods fabricated through microwave-assisted chemical synthesis. ACS Appl. Mater. Interfaces.

[19] Zhang Y., Xu J., Xu P., Zhu Y., Chen X., Yu W. (2010). Decoration of ZnO nanowires with Pt nanoparticles and their improved gas sensing and photocatalytic performance. Nanotechnology.

[20] Bora T., Kyaw H.H., Sarkar S., Pal S.K., Dutta J. (2011). Highly efficient ZnO/Au Schottky barrier dye-sensitized solar cells: Role of gold nanoparticles on the charge-transfer process. Beilstein J. Nanotechnol..

[21] Saoud K., Alsoubaihi R., Bensalah N., Bora T., Bertino M., Dutta J. (2015). Synthesis of supported silver nano-spheres on zinc oxide nanorods for visible light photocatalytic applications. Mater. Res. Bull..

[22] Khan M.N., Al-Hinai M., Al-Hinai A., Dutta J. (2014). Visible light photocatalysis of mixed phase zinc stannate/zinc oxide nanostructures precipitated at room temperature in aqueous media. Ceram. Int..

[23] Zhang Y., Gan H., Zhang G. (2011). A novel mixed-phase TiO_2_/kaolinite composites and their photocatalytic activity for degradation of organic contaminants. Chem. Eng. J..

[24] Lin X., Xing J., Wang W., Shan Z., Xu F., Huang F. (2007). Photocatalytic activities of heterojunction semiconductors Bi_2_O_3_/BaTiO_3_: A strategy for the design of efficient combined photocatalysts. J. Phys. Chem. C.

[25] Thompson T.L., Yates J.T. (2005). TiO_2_-based photocatalysis: Surface defects, oxygen and charge transfer. Top. Catal..

[26] Liu S., Li C., Yu J., Xiang Q. (2011). Improved visible-light photocatalytic activity of porous carbon self-doped ZnO nanosheet-assembled flowers. CrystEngComm.

[27] Zhang X., Qin J., Xue Y., Yu P., Zhang B., Wang L., Liu R. (2014). Effect of aspect ratio and surface defects on the photocatalytic activity of ZnO nanorods. Sci. Rep..

[28] Chen D., Wang Z., Ren T., Ding H., Yao W., Zong R., Zhu Y. (2014). Influence of defects on the photocatalytic activity of ZnO. J. Phys. Chem. C.

[29] Fang J., Fan H., Ma Y., Wang Z., Chang Q. (2015). Surface defects control for ZnO nanorods synthesized by quenching and their anti-recombination in photocatalysis. Appl. Surf. Sci..

[30] Desai U.V., Xu C., Wu J., Gao D. (2012). Solid-state dye-sensitized solar cells based on ordered ZnO nanowire arrays. Nanotechnology.

[31] Kang D., Liu A., Bian J., Sang Y. (2012). Optoelectronic characteristics of zinc oxide nanorods/P3HT hybrid junctions investigated using surface photovoltage method. Solid State Lett..

[32] Janotti A., Van de Walle C.G. (2007). Native point defects in ZnO. Phys. Rev. B.

[33] Ye J.D., Gu S.L., Qin F., Zhu S.M., Liu S.M., Zhou X., Liu W., Hu L.Q., Zhang R., Shi Y. (2005). Correlation between green luminescence and morphology evolution of ZnO films. Appl. Phys. A.

[34] Erhart P., Albe K. (2006). First-principles study of migration mechanisms and diffusion of oxygen in zinc oxide. Phys. Rev. B.

[35] Chen M., Wang X., Yu Y.H., Pei Z.L., Bai X.D., Sun C., Huang R.F., Wen L.S. (2000). X-ray photoelectron spectroscopy and auger electron spectroscopy studies of Al-doped ZnO films. Appl. Surf. Sci..

[36] Major S., Kumar S., Bhatnagar M., Chopra K.L. (1986). Effect of hydrogen plasma treatment on transparent conducting oxides. Appl. Phys. Lett..

[37] Hsieh P.T., Chen Y.C., Kao K.S., Wang C.M. (2008). Luminescence mechanism of ZnO thin film investigated by XPS measurement. Appl. Phys. A.

[38] Szörényi T., Laude L.D., Bertoti I., Kantor Z., Geretovszky Z. (1995). Excimer laser processing of indium-tin-oxide films: An optical investigation. J. Appl. Phys..

[39] Wei S., Lian J., Wu H. (2010). Annealing effect on the photoluminescence properties of ZnO nanorod array prepared by a PLD-assistant wet chemical method. Mater. Charact..

[40] Wei X.Q., Zhang Z.G., Liu M., Chen C.S., Sun G., Xue C.S., Zhuang H.Z., Man B.Y. (2007). Annealing effect on the microstructure and photoluminescence of ZnO thin films. Mater. Chem. Phys..

[41] Baruah S., Sinha S.S., Ghosh B., Pal S.K., Raychaudhuri A.K., Dutta J. (2009). Photoreactivity of ZnO nanoparticles in visible light: Effect of surface states on electron transfer reaction. J. Appl. Phys..

[42] Kavitha M.K., Jinesh K.B., Philip R., Gopinath P., John H. (2014). Defect engineering in ZnO nanocones for visible photoconductivity and nonlinear absorption. Phys. Chem. Chem. Phys..

[43] Al-Hamdi A.M., Sillanpää M., Dutta J. (2016). Intermediate formation during photodegradation of phenol using lanthanum doped tin dioxide nanoparticles. Res. Chem. Intermed..

[44] Baruah S., Mahmood M.A., Myint M.T.Z., Bora T., Dutta J. (2010). Enhanced visible light photocatalysis through fast crystallization of zinc oxide nanorods. Beilstein J. Nanotechnol..

[45] Baruah S., Dutta J. (2009). Hydrothermal growth of ZnO nanostructures. Sci. Technol. Adv. Mater..

